# Engaging citizens living in vulnerable circumstances in research: a narrative review using a systematic search

**DOI:** 10.1186/s40900-021-00306-w

**Published:** 2021-09-03

**Authors:** N. S. Goedhart, C. A. C. M. Pittens, S. Tončinić, T. Zuiderent-Jerak, C. Dedding, J. E. W. Broerse

**Affiliations:** 1 Athena Institute, Faculty of Science, Vrije Universiteit Amsterdam, Amsterdam, The Netherlands; 2 Department of Ethics, Law and Humanities, Amsterdam UMC, Amsterdam, The Netherlands

**Keywords:** Narrative review, Public engagement, Patient engagement, Citizens living in vulnerable circumstances, Socioeconomic inequalities, Health inequalities, Socioecological model, Methods

## Abstract

Although public engagement in research is increasingly popular, the involvement of citizens living in vulnerable circumstances is rarely realized. This narrative review aims to describe and critically analyse concerns and corresponding strategies, tools, and methods that could support the inclusion of these citizens in health research. The 40 studies that are included were thematically analysed using the socioecological model. Concerns originate most often on the intrapersonal level of the socioecological model, but concerns were also identified at institutional, community, and policy levels. It is thought-provoking that there is a lack of attention for the research and policy structure in which engagement practices are designed, implemented and evaluated. More research is needed to explore how these cultures could be changed in a way that promotes rather than restrains the engagement of citizens living in vulnerable circumstances in research and policymaking.

**Supplementary Information:**

The online version contains supplementary material available at 10.1186/s40900-021-00306-w.

## Plain English summary

Unfortunately, citizens living in vulnerable circumstances are seldom engaged in research or policymaking. Think of, citizens living in poverty, those with an ethnic minority background or citizens with mental health issues are often excluded. Their involvement is, however, crucial to prevent growing (health) inequalities. To support the engagement of people living in vulnerable circumstances we conducted a literature review. We looked for strategies, tools, and methods that could support the inclusion of these citizens in research or policymaking. In total, 40 studies were identified and analysed. The main finding is that strategies tools, and methods are always context depended. Researchers should always ask: What works for whom in which context? We, therefore, stress the importance of close collaboration with experienced owners and community based organisations from the start. Also, more attention should be paid to how engagement practices are designed, implemented and evaluated. Think of, strict deadlines which limit the time to build trust. Or low budgets that limit the options to create the right preconditions, think of recruitment and data collection in multiple languages. To make sure that not only the ‘usual suspects’ but also citizens living in vulnerable circumstance will be engaged attention is needed from advocacy groups, research funders and researchers. All these actors need to reflect more often on the question what they can do to change the current research and policymaking culture in such a way that it will support the engagement of citizens living in vulnerable circumstances.

**Supplementary Information:**

The online version contains supplementary material available at 10.1186/s40900-021-00306-w.

## Introduction: engaging citizens living in vulnerable circumstances in research

Public engagement in health research and policymaking is increasingly popular in Western societies. Policy, patient, and carer advocacy groups, academic communities, and funding agencies increasingly emphasize that health research does not always meet citizens’ needs and priorities [37]. By including the experiential knowledge of citizens, research becomes more contextualized and needs oriented, and thereby its quality and relevance may improve [15, 21]. Furthermore, research that is co-produced with citizens may have a broader social impact [34, 62]. Moreover, citizens and patients increasingly demand that they have a say in research and policy that affect their lives and refuse to be just passive receivers, as embodied in the well-known adage ‘Nothing about us without us’ [5].

The increased popularity means that public engagement has found its way into fields and institutes which are not always acquainted with the approaches underpinning public engagement [28, 73]. In particular, the engagement of citizens living in vulnerable circumstances, such as citizens with a low socioeconomic position (SEP), those with an ethnic minority background or citizens with mental health issues, is often seen as complicated and is rarely realized [64, 72]. While the involvement of these citizens is crucial to prevent growing (health) inequalities, they are often excluded due to failures in the composition of the research, such as inclusion criteria that demand a certain level of education or the use of complicated language in invitations to participate [16, 65, 66]. Moreover, there is a lack of awareness that our research standards and methodologies are not (culturally) neutral and value-free. Frequently used engagement formats such as advisory boards or questionnaires are more in line with the daily practices of health-care professionals, policymakers, and more highly educated citizens than with the lives of citizens living in vulnerable circumstances [43, 65, 72]. Also, public or citizen engagement is frequently used without acknowledging that communities consist of multiple groups with different values, norms, and perspectives [57].

Guidance on *how* to engage citizens living in vulnerable circumstances in research is often limited. Innovative and inclusive research practices seem to be rarely published or hard to find, so it is difficult for researchers to learn from them [57, 67]. An exception is the recent narrative review by Greenhalgh et al. [36], they explored 65 frameworks from 10 different countries, including toolkits, checklists, and benchmarks or maps for informing, guiding, assessing, or reporting on public and patient involvement (PPI). Only five frameworks (partly) provided guidance on the inclusion of hard-to-reach groups. These studies aimed to encourage the engagement of addicts and homeless people [61], diverse populations, including patients who live in poverty [83] or experience health disparities [45], and those with an ethnic minority background [7, 18]. Although this small number of individual guiding frameworks for specific groups exists, there is limited insight into the range of tools or methods that can be used and their advantages and disadvantages; more information about these could help researchers to learn how to engage more diverse groups in research and policymaking (cf. [57]).

This review aims to describe and critically analyse concerns and corresponding strategies, tools and methods that could support the inclusion of citizens living in vulnerable circumstances to strengthen the engagement of these citizens in research. In this narrative review with a systematic search, we do not explicitly intend to develop a one-size-fits-all framework but instead aim to provide an insight into different routes that can be taken. We acknowledge that there is no single best approach; the particular context and the characteristics of the involved citizens, the topic, and the resources will determine what is needed [29, 36].

### Citizens living in vulnerable circumstances

In this review, we have purposefully decided not to use the term vulnerable groups since we believe, in line with Walker and Fox [81], that it is the context that places these citizens in a vulnerable position. Vulnerability is not a characteristic of an individual or group. This review focuses on groups of individuals whose circumstances mean that they are often forgotten in engagement practices or are hard to reach.

According to Larkin [46], citizens can be hard to reach in relation to research due to (1) individual, unique or innate factors, (2) structural factors, (3) personal circumstances, or a combination of these factors. Individual, unique and innate factors refer to certain disabilities (i.e. physical or mental disabilities), which someone can be born with or has acquired since then [12, 46]. Structural factors refer to a person’s location in a hierarchical socio-cultural order in a particular society [13]*.* Someone’s socioeconomic position (SEP), race, ethnicity or age affects the potential for their involvement in research practices. Last, a person’s personal circumstances impact how they can be engaged in research or policymaking. Personal lifestyle choices or risky behaviour might cause underrepresentation in engagement practices, e.g. for individuals who use substances, or have experience of incarceration, or in a situation of prostitution [12].

## Study design

We systematically searched for articles. The initial search was broad; a flexible and interpretive approach to the screening process was deemed necessary to formulate a precise aim. Initial ideas were redefined through progressive focusing, which contributed to a deeper understanding of the proposed aim (cf. [11, 36]).

### Search

The initial search was executed in five databases: Cochrane, Embase, Web of Science, PubMed, and CINAHL. Different techniques and terms were used to expand and narrow searches, such as synonyms, medical subject headings (MESH), Boolean operators, and truncation. Keywords were sorted into three categories: (1) population: citizens living in vulnerable circumstances (e.g. minority groups, hard-to-research groups, citizens with a low SEP, substance users, etc.); (2) type of engagement (e.g. public involvement, patient engagement, co-production), and (3) the context of the studies (e.g. health policy, clinical trials, social care). The final search syntax is provided in Additional file 1: Search syntax.

### Study selection

The flexible and interpretive study selection was done in two phases. First, titles and abstracts were screened using two main criteria: articles that (1) describe the engagement of citizens living in vulnerable circumstances, and (2) report on the processes involved in citizens’ engagement in research. Two researchers (NG and ST) analysed the first 1100 articles individually to identify Cohen’s kappa coefficient (κ). After analysis of these articles, these two researchers had an almost perfect agreement, with more than 92% of articles rated in the same manner (κ = 0.8035), which increased the reliability of the systematic review and helped to avoid reporting errors [76]. Because of the high Cohen’s kappa coefficient, the researchers decided to continue working on analysis separately. A third researcher was involved to resolve any uncertainties (CP).

Full texts were screened for eligibility by two researchers (NG and ST) and discussed with the research team. We decided to narrow our focus during full-text screening. Although our search initially included multiple groups that live in vulnerable circumstances, we decided to only focus on groups living in vulnerable circumstances due to poverty, immigration status or ethnicity. Narrowing the selection was needed to create a more coherent and in-depth narrative. Groups were excluded, for example, if recent reviews or books about tools for and/or guidance on involving these groups were available, e.g. citizens with mental disabilities (e.g. [9, 10, 19, 35]), and children/young people [8, 29, 33]. The final inclusion and exclusion criteria are summarized in Table 1.

**Table 1:** Final set of inclusion and exclusion criteria

Criteria	Explanation
Study population	Studies were included if they report on citizens living in vulnerable circumstances caused by poverty, immigration status, or ethnicity. Although these populations may have different characteristics, i.e. language, religion, cultural assumptions, migration status, etc., what these groups have in common is that it is less likely that they will be engaged in research in the field of health and well-being and often experience discrimination, (social) exclusion, health disparities and stigmatization. Studies only reporting on the engagement of carers, representatives, advocates or staff were excluded
Type of engagement	Studies were included if citizens were consulted or involved as partners or co-researchers
Type of research	We only refer to the engagement of citizens on a collective level, such as involvement in priority setting for health and social care research, in the development of a prevention programme, or in clinical guideline development [78]
Study context	Studies were included if they report on research in the health and/or social care context. Moreover, the research should have taken place in the Western world, i.e. Europe, the United States of America (USA), Canada or Australia
Reporting	Only articles that (explicitly) reflect on their process of citizen engagement were included. Articles which only give minor details about their methods but did not include any reflection were excluded. For example, an article which highlights that the authors involved a translator and gave the citizens involved a gift card to compensate them for their time and travel expenses without explaining why they made this choice was excluded
Characteristics of the study	All peer-reviewed studies in English or Dutch published between December 2010 and December 2019 were included. Editorials, letters, commentaries, opinion pieces, theses, and reviews were excluded. Reviews were used to identify other relevant studies, however

### Data extraction and analyses

Data extraction from the 40 included studies was done in Microsoft Excel and included the following descriptive data:

- Study characteristics: author(s), year, journal, country, research aim, study approach, and research method.
- Study population: description of citizens and others involved (i.e. community organizations, policymakers).

The first author (NG) extracted the data and the two other authors (CP and ST) randomly checked the extraction.

All of the included articles were analysed by the first author (NG) through thematic analysis [14] using Atlas.ti [6]. The coding was randomly checked by the two other authors (CP and ST). Deductive and inductive coding were combined. Moreover, two critical friends with many years of experience in patient engagement, with respectively children and people with dementia, were invited to read a first draft of our manuscript to validate and deepen the analyses with their own practical experiences.

**Fig. 1:**
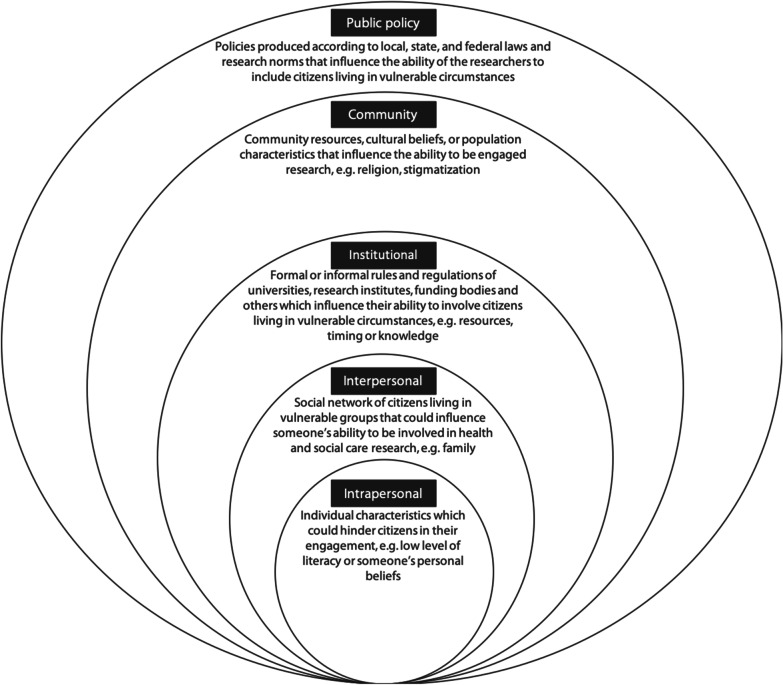
Socioecological model adopted from McLeroy [55], Daley et al. [24], and Salihu [69]

The socioecological model [55] was used as the analytical model (see Fig. 1), because in previous research, the socioecological model has been successfully applied to identify barriers to and corresponding tools/guidance for including citizens living in vulnerable circumstances in clinical trials (e.g. [24, 69]). The socioecological model provides an overview of numerous and varied factors that could influence the engagement of citizens living in vulnerable circumstances in research. Individual engagement is influenced by a dynamic set of intrapersonal characteristics, interpersonal processes, institutional factors, community features and public policies. The model assumes that there is an interaction between all levels, so the probability of an individual engaging in health and social research is influenced by their environment, but the environment is also influenced by the individual [69].

## Results

The titles and abstracts of 6782 articles were assessed. Screening the abstracts for eligibility and narrowing down the focus of this review resulted in 379 for full-text screening (Additional file 2: Flow diagram). During full-text screening many articles were excluded since they do not reflect on the method they used to engage citizens living in vulnerable circumstances (n = 158) or citizens were only involved as research subjects (n = 100). In total, 40 articles were included for in-depth analysis (Table 2).

**Table 2:** Included articles

Group	Included articles
Citizens with an ethnic minority background	Alcazar et al. [2], Belone et al. [7], Ceballos et al. [20], De Marco et al. [26], DeCamp et al. [27], Haynes-Maslow et al. [40], Irvine et al. [41], Isler et al. [42], Knifton [44], Lee et al. [47], McDavitt et al. [54], Redwood et al. [65] and Wang-Letzkus et al. [82]
Citizens who are insecurely housed	Pakhale et al. [61] and Van Draanen et al. [79]
Citizens with a migration status	Alzubaidi and Marriott [3], Alzubaidi et al. [4], Brugge et al. [17], Cyril et al. [23], de Freitas and Martin [25], Dingoyan et al. [30], Lionis et al. [48], Loignon et al. [49], O'Reilly-De Brún et al. [59, 60] Renzaho [66], Shirazi et al. [71] and Woodward-Kron et al. [84]
Citizens with a low socioeconomic position	Marinescu et al. [52] and Stewart [75]
Refugees	Haley et al. [39], Martzoukou and Burnett [53], Quinn [63] and Riggs et al. [67]
Citizens living in diverse vulnerable circumstances	Kaiser et al. [43], MacFarlane et al. [51], Montesanti et al. [57], O’Donnell et al. [58], Ryan et al. [68] and Snow et al. [72]

The articles were concerned with the inclusion of citizens with an ethnic minority background (n = 13), citizens who are insecurely housed (n = 2), citizens with migration status (n = 13), citizens with a low socioeconomic position (n = 2), or refugees (n = 4), or groups of citizens living in diverse vulnerable circumstances (n = 7) (Table 2). With the latter we refer to articles like Kaiser et al. [43] who report on engagement practices with people with challenging life experiences, including poverty, homelessness, long-term underemployment, and chronic health problems. Some articles could have been categorized as belonging in more than one of the above-mentioned groups because of the intersecting nature of the categorization. We, however, followed the descriptions given in the article. The studies were conducted in the USA (n = 17), Europe (n = 14), Canada (n = 4), and Australia (n = 6). More demographics of the included articles are shown in Additional file 3: Demographics of the included studies.

**Fig. 2:**
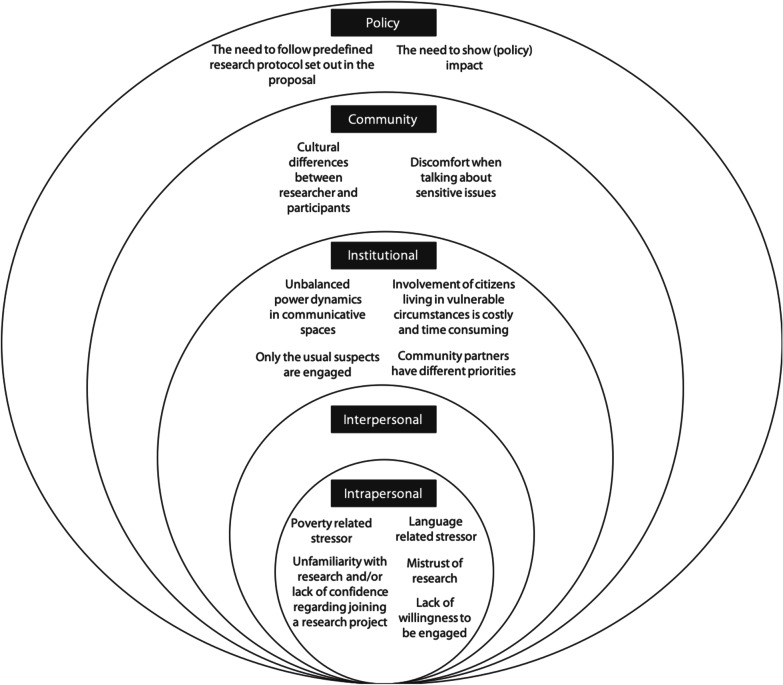
Concerns that could hinder citizens living in vulnerable circumstance from being involved in research

Concerns were identified on four levels of the socioecological model which could influence the engagement of citizens living in vulnerable circumstances in research (Fig. 2). All concerns and corresponding strategies, tools and methods are systematically summarized in Tables 3, 4 and 5. In the section below, we will explain for each level—from the intrapersonal to the policy level—the identified concerns and corresponding strategies, as well as the most often mentioned tools and methods which could be used to engage citizens living in vulnerable circumstances in research. To increase the readability the references of the identified strategies are given in Tables 3, 4 and 5 and not in the text.

Although we acknowledge that there is interaction between all levels and that consequently concerns could be linked to multiple levels, we chose to only describe the concerns and corresponding strategies, tools and methods at the level where they emerged most explicitly. And because strategies, tools and methods can be used to address multiple concerns, some of them are mentioned more than once. Some concerns or corresponding strategies, methods and tools are specific to a particular group. Where that is the case, it is made explicit in the result section. Finally, we want to emphasize that the strategies, tools, and methods described in results section are context specific and culturally sensitive and cannot be applied to each context without critical reflection.

### Intrapersonal level concerns, strategies, tools and methods

At the intrapersonal level of the socioecological model, i.e. individual characteristics that can hinder engagement in research, and corresponding strategies and tools, five concerns were identified: (1) unfamiliarity with research and/or lack of confidence regarding joining a research project, (2) lack of willingness to be engaged, (3) mistrust of research, (4) poverty-related stressors, and (5) language-related stressors (Table 3). Below we describe the strategies for each concern as identified in the articles.

**Table 3:** Concerns connected to strategies, tools and methods—intrapersonal level

Concern	Suggested strategy	Suggested tools and methods
Unfamiliarity with research [30, 42, 48, 60, 72, 75] and/or lack of confidence regarding joining a research project [25, 30, 39, 42, 48, 57, 58, 60, 68, 75]	Recruit in familiar locations or via a familiar recruiter [4, 25, 26, 30, 41, 44, 48, 51, 53, 58, 59, 60, 63, 65, 71, 72, 79, 84]	Use word-of-mouth recruitment by community (health) workers [3, 17, 26, 40, 47, 48, 51, 54, 59, 66, 67], community partners [30, 39, 40, 41, 43, 44, 48, 49, 59, 63, 68, 72, 79], family or friends [7, 65, 79, 84], bicultural researchers [54, 60, 71, 84], or someone from the network of the researcher [51, 68, 84]
		Use snowball recruitment [41, 49, 51, 75]
		Recruit at a place that people are familiar with [2, 4, 17, 23, 27, 43, 51, 71], e.g. church, CBO, school or playground, or the street, using culturally sensitive pamphlets [41, 65, 68, 75, 82]
		Make use of local news on TV or radio [17, 23, 25, 30, 63, 68, 71, 75]
	Build on the capacities and knowledge and empower those involved by offering easily accessible activities [47, 25, 48, 51]	Storyboards [59, 60, 67]
		Digital storytelling [48, 68]
		Photo-voice methodology [2]
		Theatre-related activities [25, 47]
		Unstructured dialogue with creative components [47, 57, 65, 75]
	Invite people to a familiar location [40, 47, 58, 72]	Plan the meeting at a location that participants prefer [23, 27, 30, 39, 41, 61, 63, 68, 72, 79, 84], most often identified as a familiar place, such as a café-style environment, a church, a community centre, or their own home
		Involve gatekeepers or CBOs to determine location [40, 47, 58, 72]
	Create a less threatening environment [4, 30, 39, 42, 43, 47, 51, 53, 57, 59, 60, 65, 68, 71, 72, 79, 84]	Build rapport by, for example, taking time to get to know each other [27, 42, 48, 63, 51], doing ice-breaker or team-building activities [42, 43, 53], or giving a warm welcome by offering tea and refreshments [71]
		Surround people with others who are similar [41, 63, 67, 68, 72, 84] and involve (peer) researchers or community members with the same cultural background or lived experiences [4, 60]
		Discuss (ethical) ground rules [3, 55, 63, 82]
		Do not record the meetings [30, 60, 63]
Lack of willingness to be engaged [4, 14, 20, 21, 30, 33, 36]	Think about the following question: What’s in it for them? [7, 25, 27, 30, 42, 43, 44, 54, 57, 60, 61, 63, 66, 72, 79, 82]	Maintain time for social interaction and for the participants to get to know other people involved [25, 26, 30, 43, 60, 72, 79, 82]
		Guarantee mutual learning [7, 26, 27, 30, 43, 57, 60, 61, 66, 72, 79]
		Show that you consider suggestions [27, 39, 63]
		Make sure people are being heard, and not only about the topic being discussed [82]
		Guarantee that citizens have the opportunity to make (tangible) impacts [27, 43, 54, 60, 61, 66, 72, 82] or to help other community members [7, 25, 26, 30, 42, 43, 66, 72]
	Recruit in familiar locations or via a familiar recruiter [4, 25, 26, 30, 41, 44, 48, 51, 53, 58, 59, 60, 63, 65, 71, 72, 79, 84]	See above
Mistrust of research [2, 4, 8, 11, 13, 15, 18, 21, 22, 25, 28, 31, 33, 34, 36, 38]	Involve a person who can act as a bridge between the researchers and the community to address mistrust [7, 17, 30, 48, 57, 60, 65, 79, 82, 84]	
	Recruit in familiar locations or via a familiar recruiter [4, 25, 26, 30, 41, 44, 48, 51, 53, 58, 59, 60, 63, 65, 71, 72, 79, 84]	See above; in addition:
		Be transparent about the goals and objectives [30, 43, 63, 65, 82]
		Address mistrust [7, 43, 54]
Poverty-related stressor [9, 10, 1, 34, 18, 33, 36, 30, 28, 2, 8, 14]	Pay attention to people’s daily reality by planning the activities [20, 23, 26, 42, 48, 65, 36, 72]	Accommodate (unexpected) changes in a schedule [20, 72]
	Provide non-tangible incentives which support engagement [26, 52, 61, 72]	Arrange child care [26, 30, 43, 52, 53, 57, 65]
		Provide meals and/or refreshments [26, 30, 61, 79, 52, 72]
		Offer services people cannot normally afford [61, 72]
		Pay for public transport tickets upfront [39, 65, 52, 53, 57, 75]
	Provide tangible incentives as appreciation [2, 20, 26, 30, 39, 40, 48, 51, 52, 65, 67, 68, 75, 79]	Offer cash [26, 30, 47, 57, 79]
		Offer gift cards or shopping vouchers [2, 7, 40, 52, 58, 63, 68, 71, 75, 82]
Language-related stressor: lack of proficiency in language of host country or limited literacy [1, 3, 12, 13, 16, 10, 17, 18, 20, 25, 28, 33, 34, 36, 37]	Collect data and recruit in multiple languages [4, 23, 39, 41, 44, 47, 51, 52, 53, 60, 63, 65, 68, 71, 82, 84]	Involve bilingual researchers [2, 4, 20, 26, 30, 47, 48, 51, 60, 71, 82, 84]
		Involve translators [4, 20, 23, 44, 53, 60, 65, 66, 67, 68]
		Provide information about the research in multiple languages (written and spoken) [3, 23, 30, 68, 84]
		Allow verbal consent in multiple languages [39, 40, 63, 65, 67, 68, 84]
	Create an environment in which language is less important [2, 43, 44, 47, 53, 58, 60, 65]	Storyboards [30, 59, 60]
		Drawings [51, 58] (Information world mapping approach [53]) or art [47]
		Photovoice methodology [2]
		Theatre-related activities or body mapping [25, 47]
		Unstructured dialogue with creative components [47, 65, 68, 75] in which it is safe to speak [65]

*CBO* community-based organization

#### Unfamiliarity with research and/or lack of confidence regarding joining a research project

Unfamiliarity with research and not perceiving oneself as an expert prevents some citizens from engagement; unfamiliarity might limit citizens’ confidence to share their opinions and believe that these views will be heard and acted upon (cf. [57]).

Four strategies have been found to address this concern. First, many studies highlight the need for recruitment in familiar locations and/or via a familiar recruiter to mobilize citizens who are unfamiliar with research. Word-of-mouth recruitment is most often mentioned and preferably should be done by community partners, volunteers, family or friends, or peers. For instance, De Freitas and Martin ([25] p. 8) make the following point:

Without a direct invitation by Project Apoio’s coordinator, many participants would have stayed inactive. Invitations to participate are experienced as a ‘vote of confidence’ in their personal competences and ability to make a difference*.*

Second, four articles mention that to support citizens with low self-esteem and low confidence, it is essential to build on the capacities and knowledge of those involved by offering easily accessible activities. These activities are usually creative methods through which citizens are empowered to express their voices in a group, e.g. digital storytelling, photo-voice methodology, drafting storyboards, theatre-related activities, or an unstructured dialogue with creative components. For example, Redwood et al. [65] note that creating rangoli designs—a popular South Asian art form—with ground rice was a familiar activity which empowered South Asian women with low literacy levels to have an unstructured dialogue at the intersection of culture, health, food, and faith.

Third, many articles highlight that it is essential to invite citizens to a familiar location. For example, Snow et al. [72] note that a familiar location makes citizens feel at ease and empowered. It was found that it was important to discuss with the potential participants preferences for where the interview, focus group or meeting should take place. For instance, in many studies, a participant’s home is indicated as a familiar location, but Dingoyan et al. ([30], p. 7) show this is not always the case: *“They [Individuals with Turkish migration backgrounds living in Germany] reported a fear of attack and being robbed if they allowed the alleged interviewer to enter their home.”.*

The last strategy that is mentioned is the importance of creating a less threatening environment to involve citizens who are not familiar with research. Eight articles emphasize that time is needed to build rapport, and in five other articles it is indicated that it is beneficial to surround people with similar others. This may mean that researchers have to divert from conventional quality standards. Although recording interviews or focus groups is preferred by researchers, asking for permission to do so could lead to uncertainties or inhibitions in open discussion, as explained by Dingoyan et al. ([30], p. 8), for example:

[M]any participants had concerns about the research staff’s expectations of them and whether they would be able to meet them. [...] some participants also reported concerns of being examined and providing incorrect answers. For these reasons, it seems adequate not to have audio and video taped the participants, so as not to excite their fears of being controlled or examined.

#### Lack of willingness to be engaged

Only a few studies explicitly state that some people are not motivated or just do not have time to participate. Lack of motivation or time intersect with socioeconomic distress, which causes people to dedicate their time and energy to other more important issues, as was explained by a participant in the study of Belone et al. ([7], p. 123):

We’re always in survival mode how do we get food on the table, how do we clothe ourselves, how do we get our homes? Those basic processes—just be able to survive—a lot of community are in that mode, so they’re not able to go beyond that some days.

Two strategies were identified to support these types of citizens. In 18 articles it is concluded that it is always important to keep in mind the question ‘What is in it for them?’. Wang-Letzkus et al. ([82], p. 258) note that:

[C]ommunity members showed their interest and enthusiasm for the study after being convinced that the researcher was not simply using them to conduct a research study, but rather the researcher was aiming to conduct the study with and for them.

Researchers need to make sure that involvement is worthwhile for participants by, for example, maintaining time for social interaction and getting to know the other participants, guaranteeing mutual learning, and showing that participants’ suggestions are being taken into account. Second, it is stated that recruitment in familiar locations and/or via a familiar recruiter is needed to mobilize citizens who are unfamiliar with research (as explained above), but also to mobilize those who are not motivated.

#### Mistrust of research

In many of the studied articles, it is emphasized that lack of trust in academia may hinder participants from engaging with researchers and from opening up and sharing their stories with researchers. Mistrust can be caused by negative experiences with services or research or because of damaging (or misinterpreted) stories about official authorities [30, 59, 72], services [58], or research [7, 53]. Citizens can distrust research due to historical disenfranchisement by academia, policymakers, governments, and the general population (e.g. the Tuskegee Syphilis study), and often see academics as cold strangers who use their input for their own ends [54, 61]. O’Reilly-De Brún et al. [59] warn that citizens, especially those who have experienced migration, may be afraid to join in anything official, such as research.

To address mistrust, three strategies have been identified. First, using a bridge person, which is mentioned in 11 articles. A bridge person is identified as, for instance, a same-cultural researcher, a (trained) community member or leader, or a member of staff from a community-based organization. A shared (cultural) identity and core values help to build trust, as, noted, for example, by Ceballos et al. ([20], p. 2146): “*Through their often long-standing and trusted relationships with communities, promotores provide a familiarity often unattainable by researchers, especially among underserved communities.”.*

Second, it is again highlighted that recruitment in familiar locations and/or via a familiar recruiter is essential. Third, it is important to create a less threatening environment. In addition to what is explained above, McDavitt et al. ([54], p. 38) show the importance of addressing mistrust during meetings:

[T]he CAB [community advisory board] suggested that the primary presenter address mistrust at the beginning of the presentation by telling a story that conveyed why this area of research mattered personally. When we implemented this advice, the effect in the room was palpable, establishing a feeling of personal connection between the attendees and the speaker.

#### Poverty-related stressor

Participating in an event has costs attached to it, which could prevent or hinder citizens living in vulnerable circumstance from participating. Snow et al. [72] note that citizens can experience direct costs, such as child care or transportation costs, and opportunity costs, such as missed social or work opportunities. We found three strategies to support citizens with a low level of resources.

First, many studies advise that meetings should be planned by paying attention to people’s daily reality to support the participation of citizens who often have busy schedules because they have several part-time jobs and/or a complicated family life. Marinescu et al. [52], for example, underline that we should take into account prayer time for Somali participants, and Loignon et al. [49] add that sessions should not be planned at the end of the month, since this is often a more complex time for people with a low income. Second, the provision of tangible incentives, e.g. gift cards or cash, is described in many studies as a token of appreciation for participants’ invested time and costs. Third, non-tangible incentives, e.g. child care and/or public transport tickets, support citizens with a low level of resources so that they can participate.

#### Language-related stressor

Low literacy levels or lack of proficiency in the language of the host country may hinder engagement in research. Two strategies were found to stimulate the inclusion of people who lack proficiency in the language of the host country.

First, many articles suggest collecting data and recruiting in multiple languages by involving bilingual researchers and/or translators. To recruit those who lack proficiency in the language of the host country, research information and promotion materials should be developed in multiple languages. However, it is also stressed, by Alzubaidi and Marriott ([3], p. 925), for example, that written materials will not reach everyone: “*[F]irst-generation Arab immigrants dislike reading any written materials, even those translated into Arabic, as they are likely to have limited reading proficiency in their own language*.”. To obtain informed consent, research information should be given verbally, and gaining verbal consent is recommended to prevent the exclusion of citizens with a low level of literacy.

The second suggested strategy is to create an environment in which language is less important and everyone is enabled to express his or her view. Some researchers used drawings, photos or storyboards in focus groups or meetings to provide a direct and unobtrusive means to communicate and thereby to empower citizens with a low level of literacy to express their views.

### Interpersonal level: concerns, strategies, tools and methods

On an interpersonal level no concerns were identified in the included articles that could influence someone’s ability to be involved in research (e.g. family, friends, community centres). Concerns which can be caused by individual- and family-level interactions are usually linked to (cultural) norms and values and, therefore, addressed on a community level. Although we did not identify any concerns, that does not mean that interactions with family and/or friends do not influence citizen engagement practices. For example, family and/or friends are important for the recruitment of those who seldom share their voices in research [61, 65]

### Institutional level: concerns, strategies, tools and methods

At the institutional level, i.e. the ability of universities, research institutes, and community-based organizations (CBOs) to involve citizens living in vulnerable circumstances, four concerns were identified (Table 4): (1) involvement of ‘hard-to-reach’ citizens is costly and time-consuming, (2) community partners have different priorities, (3) the power dynamics in interactions, and (4) only the usual suspects are engaged. Below, we describe the strategies for each concern as identified in the articles.

**Table 4:** Concerns connected to strategies, tools and methods—institutional level

Concern	Suggested strategy	Suggested tools and methods
Involvement of citizens living in vulnerable circumstances is costly and time-consuming [17, 26, 42, 43, 51, 54, 59, 63, 66, 72, 79]	Make sure the funding body agrees on methodology [43, 60]	–
Community partners have different priorities [7, 17, 25, 44, 54, 82]	Build trust with community partners [63, 65, 82]	Undertake ‘shoe-leather’ research [63, 65]
		Be transparent about the goals, values, and principles of the research [54, 63]
		Make time to clarify expectations [54, 63]
		Avoid regular changes of research staff [54, 43, 82]
		Create a mutual interest [47]
		Be transparent about the resources involved [7, 27]
	Host cost-neutral activities [27, 43, 48, 54, 65]	Remunerate staff or volunteers of CBOs for their efforts [7, 27]
		Be transparent about the resources involved [7, 27]
	Collect data within the context of already planned activities [54, 63]	–
Unbalanced power dynamics in interactions [3, 7, 25, 30, 41, 43, 47, 49, 51, 54, 59, 60, 61, 65, 72, 75, 79]	Act as a facilitator rather than a decision maker [43, 51, 58, 60, 61, 72]	–
	Avoid pressure to reach a consensus [42, 51, 54]	Anonymous voting [42]
		Direct ranking exercise [23, 49, 51, 58]
	Empower citizens through adaptations of ‘mainstream’ communicative spaces [25, 60, 72]	Do not use complex terms [1, 43, 54, 61]
		Wear informal clothing [51]
		Avoid professionals’ bureaucratic concerns [25]
		(Train) community members as peer researchers or co-facilitators [42, 43, 44, 48, 52, 59, 60, 61, 63, 79]
		Support continual evaluation of the process [44]
		Involve experienced facilitators [44, 49, 51, 69]
		Have an open agenda [26, 51, 72]
		Co-generate ground rules for engagement [26, 51, 60]
	Build on the capacities and knowledge and empower those involved by offering easily accessible activities [25, 47, 48, 51]	See Table 3, in addition
		Self-recording [51, 72]
Only the usual suspects are engaged [43, 58, 72]	Create informal engagement opportunities [72]	–
	Avoid inclusion criteria [43]	–

*CBO* community-based organization

#### Involvement of ‘hard-to-reach’ citizens is costly and time-consuming

Some studies address the concern that to engage citizens living in vulnerable circumstances a greater investment is needed than involving highly educated members of the community who speak the national language [17, 72]. Extra money and time are needed to build trust and capacity [17, 42, 51, 60, 61], to make efforts to understand each other and reach a consensus [42, 61], to train bicultural researchers [67], involve a translator [72], and/or support (already) overloaded community-based organizations (i.e. health facilities, social work organizations) in disadvantaged areas [17, 66]. All above authors stress that time and resources are needed to avoid research that involves only the usual suspects. To address this concern, one strategy is mentioned: make sure the funding body agrees on the methodology and timeline to avoid tokenistic research.

#### Community partners have different priorities

Many studies show the value of close collaboration with the staff of CBOs, i.e. social workers, cultural advisors, or volunteers. However, there are often difficulties with this collaboration because employees and volunteers of CBOs are busy and often only have access to a low level of resources (e.g. [25, 26, 54]). De Marco et al. ([26], p. 182) explains: “*Community leaders have credibility and trust within the community and are very passionate about their work. Often, these community leaders wear many hats with little or no compensation.”.*

In order to support collaboration with CBOs, three strategies are mentioned. First, researchers should build trust with community partners. Even though shoe-leather research is often time-consuming and the outcomes are not always clear, it is important for building trust. According to Redwood et al. ([65], p. 8) shoe-leather research involves:

[T]ravelling in the local area and ‘walking the patch’, and consisted of meeting potential gatekeepers to appropriate women’s groups at local events and open days, attending public events, speaking to people about our work informally, meeting community workers in the field, and generally raising our profile as researchers in the community.

Moreover, it is important to build trust to avoid continual changes of research staff and to be transparent about the values and principles of the research and the practical agreements and resources involved.

Second, five studies emphasize that researchers should host cost-neutral activities to avoid burdening organizations that already lack resources. Redwood et al. ([65], p. 8) note:

We stressed repeatedly that the event was to be cost-neutral to the group or organization as there was anxiety over current and future funding. We also underlined that costs for room hire, materials, refreshments, travel and child care expenses would be met by the research team.

Third, to reduce the burden on community centres, some studies suggest having research activities within standard, already planned activities. McDavitt et al. ([54], p. 3) describe how this is more practical than planning special events and how in their study this resulted in high attendance and a way of thanking the CBOs involved:

We found that coordinators of many standing meetings were actively searching for relevant and timely content and that providing content for these meetings was a valued way of ‘giving back’ to community members who had referred participants to us.

#### Unbalances power dynamics in interactions

It is highlighted in many articles that interactions are influenced by existing power relations. De Freitas and Martin ([25], p. 32), for example, state: *“Inequalities in socio-economic status, communication skills and self-confidence may lead some—usually those already marginalized—to silence themselves.”.* Four strategies were suggested to foster equity.

First, a researcher should act as a facilitator rather than someone who makes decisions; this is noted in eight studies. This promotes trust and security. A facilitator needs to be someone who is considered as neutral and does not provide health or social services. For example, a patient participant in the study of Snow et al. ([72], p. 9) states that if the facilitator is not neutral, she would be afraid that her expression of dissatisfaction could lead to negative consequences for her health care in the future: “*If I criticize the way she’s doing her job, she’s going to look at me a different way and I’m not going to get the services that I would be before.”.*

Second, three articles suggest that researchers should encourage and value diverse perspectives and therefore should avoid pursuing the perceived need to reach a consensus. Anonymous voting or a direct ranking exercise provides equal opportunities and is, therefore, helpful in controlling dominant voices or empowering those that are often silent.

Third, there is a need for adaptations of the (unwritten) ‘rules’ of interactions. Interactions often take place in a room full of citizens who demonstrate their expertise, opinion, or input verbally, often using complex terms. Some articles suggest using several practical tools to make interactions more in line with the needs of the community, such as avoiding jargon, wearing informal clothing, avoiding dealing with bureaucratic concerns, and having meetings in a location familiar to the community members. In addition, several articles mention the benefit of involving an experienced facilitator or a (trained) community member as a facilitator. Also, co-generated ground rules and open agendas are described as beneficial. This is explained by Snow et al. ([72], p. 8):

They felt that by selecting engagement issues, planners might miss the actual issues that were important to patients. By having full control of the agenda, planners may leave little space for patients to share what really matters to them

The fourth strategy is building on the capacities and knowledge and empowering those who are hard to reach by offering familiar activities, which is important when there is a diverse group with existing power dynamics. Self-recording can help citizens to express their voice, as noted by Snow et al. [72] and MacFarlane et al. [51]. In MacFarlane et al.’s [51] study, participants had the opportunity to write or draw on a tablecloth to make sure that less confident participants did not need to articulate or defend their opinion in the group.

#### Only usual suspects are engaged

Three studies highlight the concern that by recruiting citizens living in vulnerable circumstances through health-care organizations, or gatekeeper organizations, individuals who are most interested in participation are being reached out to (again) rather than those who are not in contact with any (in)formal organization [43, 58, 72]. Snow et al. [72] suggest that informal engagement opportunities need to be created to secure a more diverse input. In addition, Kaiser et al. [43] highlight that having no inclusion criteria will also result in more diversity.

### Community level: concerns, strategies, tools and methods

On the community level, i.e. community resources, cultural beliefs, or other population characteristics that could influence whether individuals are engaged in research, just two concerns were identified (Table 5): (1) cultural differences between researchers and community members, and (2) discomfort when talking about sensitive issues

**Table 5:** Concerns connected to strategies, tools and methods—community level

Concern	Suggested strategy	Suggested tools and methods
Cultural differences between researchers and participants [4, 7, 30, 39, 47, 52, 57, 60, 63, 65, 82]	Involve a same-cultural researcher [4, 20, 23, 41, 42, 54, 60, 63, 65, 71, 82]	Train same-cultural researchers (e.g. peers or students) [17, 20, 23, 54, 57, 60, 63, 65, 66, 79, 82]
	Involve community members, i.e. social workers [3], health-care professionals [4, 53, 63], bicultural experts [67], CBOs [27, 47, 48, 52, 54, 63, 65, 66], gatekeepers [67], or lay-community group members [3, 20, 39, 42, 44, 48, 51, 54, 59, 60, 61, 63, 71, 79, 82]	Let community members give feedback on protocol [3, 4, 20, 30, 39, 44, 48, 51, 54, 57, 58, 60, 61, 63, 65, 66, 71, 72, 79, 82]
		Involve community members in facilitation of meetings [42, 44, 48, 52, 59, 60, 63, 79]
	Make adaptations based on gender-related cultural norms [4, 20, 30, 39, 52, 63, 65]	Gender-specific meetin [57, 63, 65]
		Same-sex researchers [4, 20, 30, 63, 65]
		Verbal consent [39, 40, 65, 84, 63, 67, 68]
	Make adaptations based on generational-related cultural norms [39, 47, 52, 82]	Age-specific meetings [39, 52]
		Create a space where mutual respect for different generations is key by establishing guidelines and agreements [47]
Discomfort when talking about sensitive issues [3, 25, 30, 63, 65, 82]	Pay attention to data-collection methods [3, 20, 44, 58, 63, 72, 75]	One-to-one interviews [3, 20, 58, 63, 72]
		Ask questions in the third person [44, 75]
	Make use of creative activities which stimulate informal conversations [47, 65]	Body mapping [47]
		Theatre-related activities [25, 47]
		Rangoli-related craft activities [65]
	Create a less threatening environment [4, 30, 39, 42, 43, 47, 51, 53, 57, 59, 60, 65, 68, 71, 72, 79, 84]	See Table 3

*CBO* community-based organization

#### Cultural differences between researchers and community members

Cultural norms, values, and traditions can impact the willingness or opportunity to participate in research. O’Reilly-de Brún et al. [59] mentions that it is difficult for a researcher to engage with a community that has a different language and culture to those of the researcher. Four strategies are suggested for this concern.

No one-size-fits-all strategy addresses cultural differences between researchers and communities. Therefore, many studies highlight the importance of including (1) a same-cultural researcher, or (2) a person from within the community to support facilitation or make the methods used more culturally sensitive. For example, Marinescu et al. ([52], p. 59) mention that

[O]ur community partners suggested, for instance, that it was important for the Somali community to have separate groups for each gender, whereas in the Vietnamese and Khmer communities it was more important to segregate the groups by age (younger and older adults*).*

Also, as Dingoyan et al. ([30], p. 8) make explicit, researchers need to be sensitive to gender-related cultural norms to avoid doing harm:

[A]ll of the focus groups mentioned that women with Turkish migration backgrounds are not allowed to invite a male interviewer into their home without the presence of a male family member. Such an invitation could lead to conflicts with the social environment of the women, such as defamations.

To meet gender-related concerns, studies suggest gender-specific meetings and to employ both female and male researchers in order to avoid some citizens being excluded.

In addition, generational-related cultural norms should be considered. Although age-specific meetings are seen as a tool that creates a space in which everyone can participate and no opinion is suppressed, sometimes age differences can support community participation. Wang et al. [82] highlight that in Chinese American culture, education is highly valued and elders enjoy telling their life experiences to a younger generation. Therefore, the involvement of young bilingual nursing students was beneficial for building rapport and creating an open discussion.

#### Discomfort when talking about sensitive issues

Talking about diseases can be taboo in some cultures. An expression that is highly valued in Chinese culture—*Bao si bu bao yu* (‘*Share happiness, not sadness*’)—might prohibit Chinese Americans from speaking about diseases publicly [82]. De Freitas and Martin [25] describe how the stigma attached to mental illness hinders Cape Verdean migrants from attending meetings about mental illness. Cultural norms or values can cause discomfort when talking about sensitive topics, Redwood et al. ([65], p. 8):

A cultural insider explained that this [Pakistani Muslim women who were difficult to access] might be due to families’ unwillingness to discuss what are deemed to be private family matters, namely the preparation and eating of food and the social and religious practices surrounding it, with strangers and for a public purpose, i.e. research.

The included articles suggest three strategies to reduce the burden of talking about sensitive issues and related taboos. First, we need to pay attention to the data-collection methods used. It is suggested that citizens are more willing to discuss private matters in a one-to-one interview. Moreover, Knifton ([44], p. 291) emphasize that it is helpful to ask questions in the third person rather than about a person self:

Questions about mental health, stigma and discrimination were asked in the third person ‘in your community’ rather than about the person or people themselves. It was felt that this would elicit more honest findings and minimize social desirability bias.

Second, creative activities, i.e. those involving references to popular theatre or rangoli-related craft activities, can help to elicit informal conversations in which citizens more easily share stories concerning sensitive topics. For instance, Lee et al. [47] used popular theatre with Cambodian women to understand health-related issues such as alcohol use and misuse and domestic violence. Third, the included articles also noted (again) the need to build a *less threatening environment*.

### Policy level: concerns, strategies, tools and methods

On the policy level, i.e. the policy of the local state, federal laws, or the research institute, two concerns were identified: (1) the need to follow the predefined research protocol set out in the proposal, and (2) the need to show (policy) impact.

#### The need to follow the predefined protocol set out in the proposal

Engagement practices are often performed within a context of predefined research questions or (policy) problems and a context in which researchers experience pressure to show impact [25, 75]. The need to follow a proposed protocol that has been submitted to get funding can limit the opportunities of citizens living in vulnerable circumstances. For example, sometimes more time is needed create the right preconditions or researcher learn along the way learn something completely different is needed included. Articles highlight, therefore, the need for flexibility: flexibility in the method and design, but planning activities is also key [49, 54, 57, 58, 72, 75]. This is noted by Montesanti et al. ([57], p. 647), for example:

In discussing their rationale for selecting the methods used in the community participation initiatives, key informants described a ‘trial by practice’ process to ‘see what works’ rather than decisions informed by prior assessment of methods. Key informants described that changes within a community such as, social dynamics or cultural practices, made it difficult to know early on which method to use.

#### The need to show policy impact

Stewart [75] adds that we should not approach public engagement from a policy perspective in which research is paid to produce ‘measurable’ evidence for policy. She used an interpretative approach in which she made space for young adults from socioeconomically deprived areas of Scotland to talk about topics relevant to them instead of a question that matched the policy problem. This approach stimulated the participation of citizens who did not see themselves as experts in a (policy) discussion that took place on their terms.

## Discussion

This narrative review aims to describe and critically analyse concerns and corresponding strategies, tools and methods that could support the engagement of citizens living in vulnerable circumstances in research.

The described strategies, tools and methods do not provide a one-size-fits-all framework, and most of the included articles emphasize that these are context dependent. Furthermore, it is stressed that each engagement practice involving citizens living in vulnerable circumstances will generate new challenges [38]. This review, therefore, emphasizes the high value of close collaboration with peer researchers, CBOs, and/or gatekeeper organizations to make engagement practices accessible for citizens living in vulnerable circumstances. The suggested strategies and corresponding tools and methods are, however, helpful for researchers as a starting point in the planning and design of engagement practices.

The strategies for enabling the engagement of citizens living in vulnerable circumstances can all be linked to the project or study context, i.e. recruitment strategies, planning, method chosen, research environment, and outcomes. These categories overlap with previous study-focused frameworks (for example those described by Greenhalgh et al. [36]. However, this review specifies practical tools that can be used in addition to these frameworks to support the engagement of citizens living in vulnerable circumstances. It is interesting that training is only described in a few of the included articles and most often in relation to training citizens to become peer researchers [42, 43, 52, 60, 79]. In other previously published frameworks, training is also described as a way of participants gaining skills or becoming experienced with well-known research methods in order to participate in involvement practices [9, 36]. In other words, this review emphasizes the need for researchers to adapt their practices to the needs of citizens living in vulnerable circumstances rather than training citizens to become familiar with their methods.

The tools that are described help to include citizens living in vulnerable circumstances, but this does not mean that these tools are not valuable in engagement processes involving other groups. For example, this review emphasizes the added value of creative methods, such as theatre-related activities, drawing or art (e.g. [25, 47]). These creative or art-based methods are not only helpful to empower citizens living in vulnerable circumstance but are also helpful more generally to open up science to the general public [32].

### Implications for engagement practices and further research

Interestingly, many concerns originate on the intrapersonal level of the socioecological model, and relatively fewer on the institutional, community, and policy levels. In addition, most concerns were articulated from the perspectives of the citizens living in vulnerable circumstances. One explanation could be that the included articles merely focused on what individuals need to engage in research, thereby creating a misbalanced overview of concerns and related strategies. Another explanation could be that there is more to be gained for the successful involvement of citizens living in vulnerable circumstances in their one on one approaches. The concerns on ‘higher’ levels are not less important, but more could be learned on a more individual level. It is, however, thought-provoking that in the included articles there is hardly any reflection on the research and policy culture in which the researchers operate. The reflections of Pakhale et al. [61] and O’Reilly-de Brún et al. [59] are notable: they indicate that the time concern is only relevant for academics who consider the implications of time spent for academic tenure or project deadlines. Involved community members rarely emphasize that *their* invested time is an over-commitment. From their perspectives, if the problems being studied are current and complex, this implies that time is needed to address them. Just as research carried out into how the culture, structure and practices of health-care systems need to be changed to involve patients in research (cf. [70, 80]), more attention should now be paid to the changes needed in health research systems to create sustainable opportunities for citizens living in vulnerable circumstances to make their voice heard.

In addition, how citizen engagement is evaluated and funded impacts researchers’ latitude regarding engagement practices [73, 75, 77]. Policy expectations steer how research is evaluated [28, 73, 75]. Van Bekkum et al. [77] highlight that UK funders can determine the boundaries of researchers and often view citizen engagement as a ‘problem-solving tool for improving science’ (p. 9) rather than emphasizing the potential of more inclusive approaches driven by democratic imperatives. This is rather counterintuitive, because health inequalities and poverty are increasing in Western societies, which calls for renewed guidance for participatory research on the policy level which is more explicit regarding the values of social justice [74, 77].

This review emphasizes the need for researchers to reflect more on their own work and, in particular, to share in more detail the lessons learned in regard to engagement practices involving citizens who seldom get a say in research. During full-text screening, more than 44% of the articles were excluded because they do not reflect on the method used. Researchers’ reflections on their method and/or the limitations of the research should no longer be limited to expressing frustration that citizens living in vulnerable circumstances could not be engaged [75]. To support the engagement of citizens living in vulnerable circumstances, researcher should more often be involved in self-analyse and dare to share their lessons learned, positive or negative.

### Strength and limitations

A major strength of this review is that it gives an overview of aspects to pay attention to in engagement practices, is based on empirical studies, and is illustrated with many rich examples. We should, however, describe four limitations for consideration. First, no patients or lay persons have been involved in our review. We invited two critical friends with many years of experience in patient engagement, with respectively children and people living with dementia, to validate and deepen the analyses. However, we would recommend to continue this journey by sharing and critically discussing the findings of this review with lay-people, to see whether we have missed or misinterpreted issues or strategies. Second, the included articles mainly use a community-based participatory research approach (Additional file 3: Demographics of the included studies). Just a few included articles use a participatory action research or a participatory research approach, which might implicate that we missed strategies or tools, such as community mapping (e.g. [31]). The fact that just a few articles used a participatory (action) research approach could be because of the databases used. We used snowball technology, however, to ensure that we could potentially identify relevant articles from other databases. The third limitation is that we only included peer-reviewed articles and no book and/or grey literature, and reflections on methodologies are often published in books or grey literature since they have more space for these critical and in-depth reflections [1, 50, 56]. Fourth and last, this review was only focused on research conducted in Western countries. Lessons learned in this review might also be useful for researchers undertaking participatory studies in low and middle income countries (LMIC). More importantly, we believe insights in engagement practices learned in LMIC could be valuable for improving practices in Western countries as participatory research has a long and rich history in involving people living in vulnerable circumstances [22].

## Conclusion

This narrative review shows that there are concerns at various levels of the socioecological model—from the intrapersonal to the policy level—that could hinder engagement of citizens living in vulnerable circumstances in research. Strategies, tools and methods throughout the entire research cycle are identified, from recruitment to research execution and outcomes. Due to the context dependency of these strategies, tools and methods, this narrative review emphasizes that the involvement of peer researchers, CBOs, and/or gatekeeper organizations is key to realizing the engagement of citizens living in vulnerable circumstances.

Just as attention has been given over the years to open up science for the public by advocacy groups, research funders and researchers, attention is now needed from all these actors to ensure that not only the ‘usual suspects’ but also citizens living in vulnerable circumstances will be engaged. In order to achieve this, researchers should not only reflect on their own challenges in projects or research practices but should also reflect on the current policy and research culture, which does not support citizens living in vulnerable circumstances in a systematic way to be involved in research, and what it will take to change that context.

## Supplementary Information

**Additional file 1.** Search syntax.

**Additional file 2.** Flow diagram.

**Additional file 3.** Demographics of the included articles.

## Data Availability

Not applicable.

## Abbreviations

CBO: Community-based organization
LMIC: Low and middle income countries
MESH: Medical subject headings
PPI: Public and patient involvement
SEP: Socioeconomic position
USA: United States of America
