# Supplementary material for: Engaging citizens living in vulnerable circumstances in research: a narrative review using a systematic search
Source: Res Involv Engagem. 2021 Sep 3;7:59. doi: 10.1186/s40900-021-00306-w (PMC8414765; doi:10.1186/s40900-021-00306-w)
Supplement: Supplementary file 1 — Additional file 1. Search syntax. [file 40900_2021_306_MOESM1_ESM.pdf]

Additional file 1: Search syntax

PUBMED - Number of articles: 3435
((((("public participation"[All Fields] OR "community participation"[All Fields] OR "patient participation"[All Fields] OR "patient participation"[MeSH Terms] OR "community participation"[MeSH Terms] OR "public involvement"[All Fields] OR "community involvement"[All Fields] OR "patient involvement"[All Fields] OR "public engagement"[All Fields] OR "community engagement"[All Fields] OR "patient engagement"[All Fields] OR "Co-research"[All Fields] OR "Co-construction of knowledge"[All Fields] OR "co-design"[All Fields] OR "co-production"[All Fields]) AND ("development of medicine"[All Fields] OR "Health care" OR "health practice" OR ("research"[MeSH Terms] OR "research"[All Fields]) OR "Health research"[All Fields] OR "Clinical trials"[All Fields] OR "Health technology assessment"[All Fields] OR "Agenda setting"[All Fields] OR "Health policy"[All Fields] OR "Social care research"[All Fields] OR "Social research"[All Fields] OR "social welfare"[MeSH Terms] OR "social welfare"[All Fields] OR "Participatory research"[All Fields] OR "community-based participatory research"[MeSH Terms] OR "Community-Based Participatory Research"[All Fields]) AND ("Minority group"[All Fields] OR "Vulnerable group"[All Fields] OR "vulnerable populations"[MeSH Terms] OR "vulnerable population"[All Fields] OR Vulnerability[All Fields] OR "hard to reach"[All Fields] OR "Hidden group"[All Fields] OR "disadvantaged groups"[All Fields] OR "Silent voice"[All Fields] OR "Seldom heard"[All Fields] OR "Medically underserved"[All Fields] OR marginalized[All Fields] OR unprivileged[All Fields] OR "minority health"[MeSH Terms] OR "Minority health"[All Fields] OR "vulnerable populations"[MeSH Terms] OR "Vulnerable populations"[All Fields] OR "intellectual disability"[All Fields] OR "mental disability"[All Fields] OR "Mental illness"[All Fields] OR "cognitive impairment"[All Fields] OR "mental disease"[All Fields] OR "Ethnic minorities"[All Fields] OR ("emigrants and immigrants"[MeSH Terms] OR ("emigrants"[All Fields] AND "immigrants"[All Fields]) OR "emigrants and immigrants"[All Fields] OR "emigrants"[All Fields]) OR ("emigrants and immigrants"[MeSH Terms] OR ("emigrants"[All Fields] AND "immigrants"[All Fields]) OR "emigrants and immigrants"[All Fields] OR "immigrants"[All Fields]) OR "Asylum seekers"[All Fields] OR "refugees"[MeSH Terms] OR ("refugees"[MeSH Terms] OR "refugees"[All Fields]) OR ("child"[MeSH Terms] OR "child"[All Fields]) OR ("adolescent"[MeSH Terms] OR "adolescent"[All Fields] OR "adolescents"[All Fields]) OR Youngsters[All Fields] OR ("adolescent"[MeSH Terms] OR "adolescent"[All Fields] OR "youth"[All Fields]) OR kids[All Fields] OR ("prisoners"[MeSH Terms] OR "prisoners"[All Fields] OR "prisoner"[All Fields]) OR ("sex workers"[MeSH Terms] OR ("sex"[All Fields] AND "workers"[All Fields]) OR "sex workers"[All Fields] OR "prostitute"[All Fields]) OR "sex worker"[All Fields] OR ("alcoholics"[MeSH Terms] OR "alcoholics"[All Fields] OR "alcoholic"[All Fields]) OR "alcohol addict"[All Fields] OR "drug abuser"[All Fields] OR "drug addict"[All Fields] OR "drug users"[All Fields] OR "low socioeconomic status"[All Fields] OR "low income population"[All Fields] OR "low socioeconomic position"[All Fields] OR "poor

person"[All Fields] OR "medically uninsured"[All Fields] OR "Low health literacy"[All Fields] OR "Low literacy"[All Fields] OR ("homeless persons"[MeSH Terms] OR ("homeless"[All Fields] AND "persons"[All Fields]) OR "homeless persons"[All Fields] OR "homeless"[All Fields]) OR "alzheimer disease"[MeSH Terms] OR "Alzheimer disease"[All Fields] OR "dementia"[MeSH Terms] OR ("dementia"[MeSH Terms] OR "dementia"[All Fields])) NOT ("non-western countries"[All Fields] OR "low-income countries"[All Fields] OR "developing countries"[MeSH Terms] OR "developing countries"[All Fields] OR ("africa"[MeSH Terms] OR "africa"[All Fields]) OR "South-east Asia"[All Fields] OR "africa"[MeSH Terms]))))) Filters: Journal Article; Clinical Trial; Editorial; Systematic Reviews; Publication date from 2010/01/01
**COCHRANE - Number of articles: 492**
#1 MeSH descriptor: [Community Participation] explode all trees 1408<br>#2 "Community Participation" 433<br>#3 MeSH descriptor: [Patient Participation] explode all trees 1161<br>#4 "Public participation" 9<br>#5 "Patient Participation" 2136<br>#6 "Patient engagement" 301<br>#7 "Public engagement" 11<br>#8 "Community engagement" 180<br>#9 "co-creation" 11<br>#10 "Community involvement" 87<br>#11 "co-production" 17<br>#12 "co-construction of knowledge" 0<br>#13 "co-research" 34<br>#14 "Public involvement" 74<br>#15 "Patient involvement" 301<br>#16 #1 OR #2 OR #3 OR #4 OR #5 OR #6 OR #7 OR #8 OR #9 OR #10 OR #11 OR #12 OR #13 OR #14 OR #15 3290<br>#17 Research 433259<br>#18 "Clinical trials" 170902<br>#19 "Health Technology Assessment" 0<br>#20 "Health research" 7971<br>#21 "Agenda setting" 35<br>#22 "Health Policy" 1713<br>#23 "Social care research" 79<br>#24 "Social research" 164<br>#25 "health care" 48198

#26 "care practice" 1005

#27 MeSH descriptor: [Social Welfare] explode all trees 803

#28 "Participatory research" 466

#29 "social welfare" 479

#30 “development of medicine” 1

#31 MeSH descriptor: [Community-Based Participatory Research] explode all trees 219

#32 "community-based participatory research" 376

#33 #17 OR #18 OR #19 OR #20 OR #21 OR #22 OR #23 OR #24 OR #25 OR #26 OR #27 OR #28 OR #29 OR #30 OR #31 OR #32 560456

#34 “Minority group” 193

#35 “Vulnerable group” 174

#36 "Vulnerable Populations" 539

#37 Vulnerability 1560

#38 "Hard to reach" 218

#39 "hidden group" 2

#40 “disadvantaged groups” 126

#41 "silent voice" 0

#42 "seldom Heard" 0

#43 "Medically underserved" 352

#44 marginalized 157

#45 unprivilaged 0

#46 "minority health"109

#47 MeSH descriptor: [Minority Health] explode all trees 21

#48 "Vulnerable populations" 539

#49 "intellectual disability" 1048

#50 "mental disability" 50

#51 “Mental illness” 2488

#52 "mental disease" 3483

#53 “Ethnic minorities” 428

#54 emigrants 176

#55 immigrants 407

#56 “Asylum seekers” 25

#57 MeSH descriptor: [Refugees] explode all trees 91

#58 "refugees" 237

#59 Child 128954

#60 Adolescents 15886

#61 Youngsters 127

#62 Youth 5178

#63 Kids 845

#64 Prisoner 183

#65 Prostitutes 28

#66 “sex workers” 271

#67 alcoholic 4637

#68 “alcohol addicts” 14

#69 “drug abusers” 165

#70 “drug addicts” 60

#71 "drug users" 928

#72 “low socioeconomic status” 385

#73 “low income population” 97

#74 “low socioeconomic position” 8

#75 "poor person" 2

#76 “medically uninsured” 168

#77 “Low health literacy” 150

#78 “Low literacy” 256

#79 homeless 707

#80 MeSH descriptor: [Alzheimer Disease] explode all trees 2922

#81 "alzheimer Disease" 8354

#82 MeSH descriptor: [Dementia] explode all trees 5010

#83 "dementia" 18302

#84 “cognitive impairment” 5910

#85 #34 OR #35 OR #36 OR #37 OR #38 OR #39 OR #40 OR #41 OR #42 OR #43 OR #44 OR #45 OR #46 OR #47 OR #48 OR #49 OR #50 OR #51 OR #52 OR #53 OR #54 OR #55 OR #56 OR #57 OR #58 OR #59 OR #60 OR #61 OR #62 OR #63 OR #64 OR #65 OR #66 OR #67 OR #68 OR #69 OR #70 OR #71 OR #72 OR #73 OR #74 OR #75 OR #76 OR #77 OR #78 OR #79 OR #80 OR #81 OR #82 OR #83 OR #84 172123

#86 "non-western countries" 28

#87 "low income countries" 871

#88 MeSH descriptor: [Developing Countries] explode all trees 799

#89 "Developing countries" 3539

#90 MeSH descriptor: [Africa] explode all trees 6124

#91 Africa 8265

#92	"south east asia" 220
#93	#86 OR #87 OR #88 OR #89 OR #90 OR #91 OR #92 15464
#94	#16 AND #33 AND #85 NOT #93 with Cochrane Library publication date from Jan 2010 to present

EMBASE - Number of articles: 3109

('community participation'/exp OR 'community participation' OR 'community involvement' OR 'community engagement'/exp OR 'community engagement' OR 'patient participation'/exp OR 'patient participation' OR 'patient engagement'/exp OR 'patient engagement' OR 'patient involvement'/exp OR 'patient involvement' OR 'public engagement'/exp OR 'public engagement' OR 'public participation'/exp OR 'public participation' OR 'public involvement' OR 'co-creation' OR 'co-research' OR 'co-construction of knowledge' OR 'co-production') AND ('participatory research'/exp OR 'participatory research' OR 'community-based participatory research' OR 'health care'/exp OR 'health care' OR 'health care practice'/exp OR 'health care practice' OR 'research'/exp OR 'research' OR 'health research' OR 'clinical trial'/exp OR 'clinical trial' OR 'health technology assessment' OR 'agenda setting' OR 'health care policy'/exp OR 'health care policy' OR 'social care research' OR 'social research'/exp OR 'social research' OR 'social welfare'/exp OR 'social welfare') AND ('vulnerable population'/exp OR 'vulnerable population' OR 'vulnerability'/exp OR 'vulnerability' OR 'medically underserved'/exp OR 'medically underserved' OR 'mental disease'/exp OR 'mental disease' OR 'immigrants'/exp OR 'immigrants' OR 'refugee'/exp OR 'refugee' OR 'child'/exp OR 'child' OR 'adolescent'/exp OR 'adolescent' OR 'prisoner'/exp OR 'prisoner' OR 'sex worker'/exp OR 'sex worker' OR 'homeless person'/exp OR 'homeless person' OR 'alzheimer disease'/exp OR 'alzheimer disease' OR 'dementia'/exp OR 'dementia' OR 'minority group' OR 'vulnerable group' OR 'hard to reach' OR 'hidden group' OR 'disadvantaged group' OR 'silent voice' OR 'seldom heard' OR 'marginalized' OR 'unprivilaged' OR 'minority health' OR 'mental illness' OR 'mental disability' OR 'intellectual disability' OR 'ethnic minority' OR 'emigrants' OR 'asylum seekers' OR 'youngsters' OR 'youth' OR 'kids' OR 'prostitutes' OR 'alcoholic' OR 'alcohol addicts' OR 'drug abusers' OR 'drug addicts' OR 'drug users' OR 'low socioeconomic status' OR 'low income population' OR 'low socioeconomic position' OR 'poor persons' OR 'medically uninsured' OR 'low health literacy' OR 'low literacy' OR 'cognitive impairment') NOT ('low income country' OR 'developing country'/exp OR 'developing country' OR 'africa'/exp OR 'africa' OR 'southeast asia' OR 'non western country') AND ([article]/lim OR [conference paper]/lim OR [editorial]/lim OR [review]/lim) AND ([dutch]/lim OR [english]/lim) AND [embase]/lim AND [2010-2019]/py

CINAHL - Number of articles: 673

(("public engagement" OR "public involvement" OR "public participation" OR "patient engagement" OR "patient involvement" OR "community engagement" OR "patient participation" OR "community involvement" OR "community participation" OR "co-research" OR "co-construction of knowledge" OR "co-production" OR "co-creation")) AND (((MH "Research") OR "research" OR "development of medicine" OR "community based participatory research" OR "participatory research" OR (MH "Social Welfare") OR "social

welfare" OR "Social research" OR "Social care research" OR (MH "Health Policy") OR "Health policy" OR "Agenda Setting" OR "Health technology assessment" OR (MH "Clinical Trials") OR "clinical trials" OR "health research" OR "health care" OR "care practice")) AND (("Minorit* group" OR (MH "Minority Groups") OR "Vulnerable group" OR (MH "Vulnerability") OR "Vulnerable populations" OR "hard to reach" OR "Hidden group" OR "disadvantaged groups" OR "Silent voice" OR "Seldom heard" OR (MH "Medically Underserved") OR "Medically underserved" OR "marginalized" OR "unprivileged" OR "Minority health" OR (MH "Mental Disorders") OR "mental disabilit*" OR "Mental illness" OR "Mental disease" OR (MH "Intellectual Disability") OR "Intellectual disability" OR "Ethnic minorities" OR "emigrants" OR (MH "Immigrants") OR "immigrants" OR "Asylum seekers" OR (MH "Refugees") OR (MH "Child") OR "Child*" OR "Adolescents" OR "Youngsters" OR "Youth" OR "kids" OR (MH "Dementia") OR "Dementia" OR (MH "Alzheimer's Disease") OR "cognitive impairment" OR (MH "Homeless Persons") OR "Homeless" OR "Low literacy" OR "Low health literacy" OR (MH "Medically Uninsured") OR "medically uninsured" OR "poor person" OR "low socioeconomic position" OR "low income population" OR "low socioeconomic status" OR "drug users" OR "drug addicts" OR "drug abusers" OR "alcohol addicts" OR "alcoholic" OR "sex workers" OR "prostitutes" OR (MH "Prisoners") OR "prisoner")) NOT (("non-western countries" OR (MH "Developing Countries") OR "developing countries" OR "low income countries" OR (MH "Africa") OR "africa" OR "south east asia"))<br>Limiters - Published Date: 20100101-; Language: Dutch/Flemish, English; Search modes - Boolean/Phrase
**WEB OF SCIENCE - Number of articles: 1060**
((((TS= ("community participation" OR "community involvement" OR "co-creation" OR "patient engagement" OR "community engagement" OR "patient participation" OR "public participation" OR "public involvement" OR "patient involvement" OR "public engagement" OR "co-research" OR "co-construction of knowledge" OR "Co-production")) AND (TS=("development of medicine" OR "research" OR "clinical trials" OR "health research" OR "Health technology assessment" OR "Agenda Setting" OR "health policy" OR "Social care research" OR "social research" OR "social welfare" OR "participatory research" OR "community based participatory research" OR "health care" OR "care practice")) AND (TS=("Minority group" OR "Vulnerable group" OR "vulnerable population" OR "Vulnerability" OR "hard to reach" OR "Hidden group" OR "disadvantaged groups" OR "Silent voice" OR "Seldom heard" OR "Medically underserved" OR "marginalized" OR "unprivileged" OR "Minority health" OR "Vulnerable populations" OR "intellectual disability" OR "mental disability" OR "Mental illness" OR "cognitive impairment" OR "mental disease" OR "Ethnic minorities" OR "emigrants" OR "immigrants" OR "Asylum seekers" OR "Refugees" OR "Child" OR "Adolescents" OR "Youngsters" OR "Youth" OR "kids" OR "prisoner" OR "prostitute" OR "sex worker" OR "alcoholic" OR "alcohol addict" OR "drug abuser" OR "drug addict" OR "drug users" OR "low socioeconomic status" OR "low income population" OR "low socioeconomic position" OR "poor person" OR "medically uninsured" OR "Low health literacy" OR "Low literacy" OR "homeless" OR "Alzheimer disease" OR "dementia")) NOT (TS=("developing countries" OR "non-western countries" OR "low income countries" OR

"africa" OR "south east Asia"))))) AND LANGUAGE: (English OR Dutch) AND DOCUMENT TYPES: (Article OR Editorial Material OR Review)

Indexes=SCI-EXPANDED, SSCI, A&HCI, ESCI Timespan=2010-2018
