# Supplementary figures and images for: Engaging citizens living in vulnerable circumstances in research: a narrative review using a systematic search
Source: Res Involv Engagem. 2021 Sep 3;7:59. doi: 10.1186/s40900-021-00306-w (PMC8414765; doi:10.1186/s40900-021-00306-w)

## Additional file 2: Flow diagram

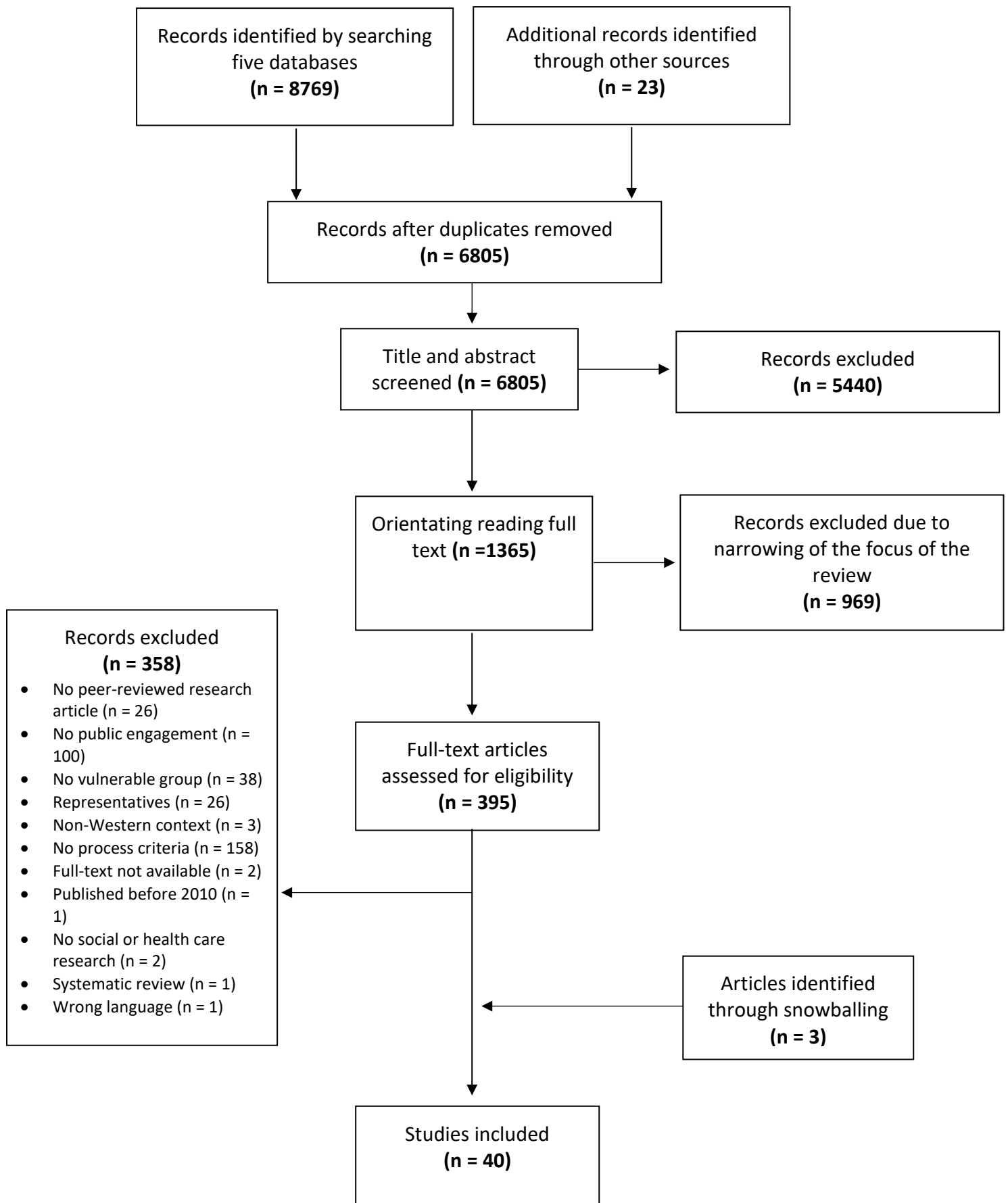

Supplement: Supplementary file 2 — Additional file 2. Flow diagram. [file 40900_2021_306_MOESM2_ESM.pdf]
