# Supplementary material for: Engaging citizens living in vulnerable circumstances in research: a narrative review using a systematic search
Source: Res Involv Engagem. 2021 Sep 3;7:59. doi: 10.1186/s40900-021-00306-w (PMC8414765; doi:10.1186/s40900-021-00306-w)
Supplement: Supplementary file 3 — Additional file 3. Demographics of the included articles. [file 40900_2021_306_MOESM3_ESM.pdf]

Additional file 3: Demographics of the included articles

Citizens with an ethnic minority background (n=13)					
Reference	Country	Aim	Study approach	Study method	Citizens involved
Alcazar et al. (2017) [2]	USA	To explore the impact of the Brighter Bites programme from the perspective of low-income Spanish families using photovoice.	CBPR	Photovoice	Parents of low-income Spanish-speaking families
Belone et al. (2016) [7]	USA	To improve the conceptual CBPR model.	CBPR	Focus groups	African American, American Indian, Asian, Latino, and White
Ceballos et al. (2015) [20]	USA	To describe the development of a culturally appropriate support group and to report on the feasibility of implementation and preliminary outcomes.	Mixed method study	Focus groups, interviews, questionnaires	Bilingual/bicultural Latinos
DeCamp et al. (2015) [27]	USA	To describe the process of establishing a family advisory board composed of Latino families at a paediatric primary care clinic, with a focus on initial partnership development and the experiences of family members on the board.	Multimodal evaluation of advisory board	Observations, member check-ins, group reflection session, and semi-structured interviews	Low-income Latino families with limited English proficiency
De Marco et al. (2014) [26]	USA	To describe the process of establishing a relationship between a research university and a Black church in rural North Carolina.	CBPR	Gardening	Visitors to a Black church
Haynes-Maslow et al. (2014) [40]	USA	To explore why African American women have low enrolment rates in clinical trials.	Qualitative research approach	Focus groups	African Americans

Irvine et al. (2017) [41]	UK	To listen to people with Chinese backgrounds about their experiences of personalization.	Phenomenology	Semi-structured interviews and focus groups	UK residents with a Chinese background
Isler et al. (2015) [42]	USA	To describe a CBPR approach with Black youths aged 18 to 30.	CBPR	Research Literacy Workgroup group discussions on a regular basis	Black Americans
Knifton (2012) [44]	Scotland	To explore mental health stigma with the three largest ethnic minority communities (Pakistani, Chinese and Indian ethnic minority) groups in Scotland.	CBPR	Focus groups	Minority communities with a Pakistani, Chinese, or Indian heritage
Lee et al. (2016) [47]	USA	To design a pilot intervention and develop the leadership capacity of grassroots community members based on their own experiences with behavioural health issues.	CBPR	Biweekly meetings in which creative methods are used	Cambodian American women, Cambodian-born refugees, American-born children of refugees, immigrants from Cambodia
McDavitt et al. (2016) [54]	USA	To identify strategies for facilitating two-way dialogue and the development of trust with communities to gain a better understanding of the findings and to explore the implications of culturally relevant interventions and public policy.	CBPR	Participatory observations and evaluation meetings with community advisory board	Black men and women living with HIV
Redwood et al. (2012) [65]	UK	To explore the intersections of faith, culture, health, and food to generate evidence for addressing cultural difference in the design and provision of health services.	Qualitative research approach	Rangoli workshop	South Asian women
Wang-Letzkus et al. (2012) [82]	USA	To share culturally competent strategies and lessons learned from a	CBPR	Focus groups, questionnaires,	Chinese American elderly people

		study that used a CBPR approach with elderly diabetic Chinese Americans.		meetings, and presentations with CAB	
Citizens who are insecurely housed (n=2)					
Reference	Country		Study approach	Study method	Citizens involved
Van Draanen et al. (2013) [79]	Canada	To investigate the factors that facilitated the meaningful inclusion of Caucus members in research and service planning from the perspectives of Caucus members and project stakeholders.	Qualitative research approach	Document review, interviews, focus groups	Caucasian members, people with lived experience of homelessness and/or mental health issues
Pakhale et al. (2016) [61]	USA	To describe the processes utilized in citizen or patient engagement research through a tobacco dependence management project with the inner-city population in Ottawa, Canada.	CBPR	Weekly meetings with peer researchers	Community members with current or past experience of drug use, current or ex-tobacco smokers who are/were homeless or insecurely housed
Citizens with a migration status (n=13)					
Reference	Country		Study approach	Study method	Citizens involved
Alzubaidi and Marriott (2014) [3]	Australia	To investigate Arabic-speaking immigrants' views and beliefs about diabetes, the use of medicines, health care-seeking behaviours, and their needs and preferences.	PPI	Interviews and semi-structured interviews	Arabic-speaking immigrants
Alzubaidi et al. (2017) [4]	Australia	To explore preferences among Arabic-speaking migrants with type 2 diabetes in Melbourne, Australia.	PPI	Interviews and semi-structured interviews	Arabic-speaking immigrants
Brugge et al. (2010) [17]	USA	To compare three case examples of CBPR that differ in terms of community and involvement.	Qualitative comparative analyses	Secondary document analysis	(1) Chinese immigrants living in or attending school or clinics in Boston's Chinatown, (2) Public housing residents, including immigrant residents, (3)

					community members of Boston's Chinatown
Cyril et al. (2017) [23]	Australia	To explore the barriers to and facilitators of the participation of culturally and linguistically diverse communities in the existing obesity prevention services in Victoria, Australia.	Qualitative research approach	Nominal group technique	Vietnamese, Burmese, African, Afghani, and Indian Australians living in disadvantaged areas
De Freitas and Martin (2015) [25]	The Netherlands	To examine the factors that influence minority service-users' decisions to get involved and stay engaged through a study of a successful mental health advocacy project hosted by a Dutch user organization.	Qualitative research approach	Interviews, observations, document analysis	Cape Verdean migrants
Dingoyan et al. (2012) [30]	Germany	To provide information about attitudes towards health research studies as well as to identify potential barriers to and solutions for the successful recruitment of individuals with Turkish migration backgrounds into health research studies.	Qualitative research approach	Focus groups	German residents with a Turkish migration background
Loignon et al. (2018) [48]	Canada	To support knowledge co-construction and translation with respect to frontline care and services by engaging underserved or Aboriginal people with low literacy skills in the research.	Participatory research	Meetings	People who are illiterate people, immigrants who have difficulty expressing themselves in French or English, and people with multi-morbidities
Lionis et al. (2016) [49]	Several European countries	To explore whether the available guidelines and training on communication regarding cross-cultural care initiatives make sense to migrants and other key stakeholders.	PLA	Focus groups	Migrants using local primary care services

O'Reilly-De Brún et al. (2015) [59]	Ireland	To reflect on the use of formal versus informal interpreters in PLA studies.	PLA	PLA-style focus groups	Migrant service users with limited English proficiency
O'Reilly-De Brún et al. (2016) [60]	Ireland	To describe PLA methodology, the mode of engagement, and techniques used for enhancing migrants' access to and engagement in the research process, and to report their evaluation of engagement.	PLA	PLA-style focus groups	Immigrants from Russia, Nigeria, Poland, Pakistan, Democratic Republic of the Congo, Portugal, Nigeria
Renzaho (2017) [66]	Australia	To develop a community engagement framework to improve childhood obesity prevention among migrants.	CBPR	African Review Panel (ARP)	Culturally and linguistically diverse migrant communities
Shirazi et al. (2013) [71]	USA	To determine the knowledge, attitudes towards, and sources of information regarding breast health care and to identify specific religious and/or social barriers for Muslim Afghan women immigrants to seeking care.	CBPR	Interviews	Immigrant Muslim Afghan women
Woodward-Kron et al. (2016) [84]	Australia	To explore: i) the perceptions and experiences of the medical system and medical research of CALD patients; ii) the views of research professionals on CALD patient participation in medical research; and iii) to inform the development of a multimedia resource for medical research and clinical trials.	Qualitative research approach	Interviews and focus groups	Older Italian migrants
Citizens with a low socioeconomic position (n=2)					
Reference	Country		Study approach	Study method	Citizens involved

Marinescu et al. (2013) (2013) [52]	USA	To describe how CBPR methods are used for developing and evaluating the Be Active Together intervention.	CBPR	Meetings and focus groups	Low-income Somali-, Vietnamese-, Khmer-, and English-speaking residents
Stewart (2015) [75]	Scotland	To understand the perspectives of young adults on local practices of public involvement in a socioeconomically deprived area of Scotland.	Qualitative research approach	Interviews	Young adults (18–25 years old) living in a socioeconomically deprived area of Scotland
Citizens living in divers vulnerable circumstances, divers (n=6)					
Reference	Country		Study approach	Study method	Citizens involved
Kaiser et al. (2017) (2016) [43]	USA	To describe the origins, participants, processes, and outcomes of a community advisory board comprised solely of lay stakeholders that brings the voices of rarely heard groups into the research enterprise.	Evaluation		People with challenging life experiences, including poverty, homelessness, long-term underemployment, and chronic health problems
MacFarlane et al. (2017) [51]	Ireland and USA	To analyse the use of World Cafés as a participatory method for research prioritization with marginalized communities in Ireland and the USA.	Comparative analysis of two cases	Observations and World Cafés	Ireland: English-speaking migrants, Irish Travellers, women who had experienced domestic violence, people with disabilities, and people living in poverty.<br>USA: Bhutanese Chinese and Karen refugees and Latin American immigrants
Montesanti et al. (2016) [57]	Canada	To better understand how health service organizations carry out participation initiatives with marginalized populations.	Qualitative comparative analyses of four cases	Key informant interviews with community health workers and secondary document analyses	(1) Low-German speaking Mennonite farming populations, (2) newcomers and immigrants, (3) elderly immigrant francophones', and (4) women who are refugees
O'Donnell et al. (2016) [58]	Ireland	To involve members of marginalized groups in the development of local	PLA	PLA-style focus groups	Migrants, homeless people, Irish Travellers, drug users, sex

		primary care services in Ireland by incorporating their views about priority areas for action.			workers, young mothers from deprived areas
Ryan et al. (2017) [68]	UK	To explore whether there are core components of good care that apply across different populations and conditions.	Qualitative research approach	Focus groups	Migrant workers, Irish Travellers, young men, illegal drug users, people with long-term health conditions, learning-disabled people, and elderly who meet for educational and cultural activities through the University of the Third Age
Snow et al. (2018) [72]	Canada	To develop a heuristic model to assist planners to engage patients who are not traditionally included in health-care planning.	Qualitative research approach	Literature review, interviews, focus groups	Women who were pregnant and/or new parenting mothers affected by substance use and/or violence; new immigrants to Canada; and Indigenous people who had a history of incarceration
Refugees (n=4)					
Reference	Country		Study approach	Study method	Citizens involved
Hayley et al. (2013) [39]	USA	To describe the process used and results derived from implementing the university/WRAP collaborative adaptation of the Eat Walk Sleep healthy living curricular tool.	Community-engaged approach	Meetings, qualitative pilot sessions, pilot workshops	Community advisors from four ethnic groups from Burma: Karen, Karenni, Kacin, and Chinese
Martzoukou and Burnett (2018) [32]	Scotland	To explore the nature of information provided to Syrian refugees and the emerging information needs and barriers they experienced during their initial phase of resettlement and adaptation in a new sociocultural environment.	Qualitative research approach	Interviews and focus groups	Syrian new to Scotland

Riggs et al. (2015) [67]	Australia	(1) To report on the steps that were taken in developing the Having a Baby in a New Country project; (2) to describe the challenges and ethical dilemmas associated with implementing community and health service engagement strategies; and (3) to reflect on lessons learned from the study.	CBPR	Community researchers: meetings, training; Community advisory group: meetings; Community: focus groups and interviews	Afghan women and men
Quinn (2013) [63]	Scotland	To investigate the different beliefs regarding and attitudes to mental health problems among the asylum seeker and refugee population in Glasgow.	PAR	Focus groups	Asylum seekers from a range of countries including Somalia, Eritrea, Pakistan, Iran, Iraq, China, and Sri Lanka

Notes: CBO = community-based organization, CBPR = community-based participatory research, PPI = public and patient involvement, PLA = participatory learning in action, PAR = participatory action research
